# Uptake of maternal care and childhood immunization among ethnic minority and Han populations in Sichuan province: a study based on the 2003, 2008 and 2013 health service surveys

**DOI:** 10.1186/s12884-019-2371-y

**Published:** 2019-07-16

**Authors:** Juying Zhang, Yuchan Mou, Jiaqiang Liao, Huaying Xiong, Zhanqi Duan, Yuan Huang, Carine Ronsmans

**Affiliations:** 1 Department of Epidemiology and Biostatistics, West China School of Public Health and West China Fourth Hospital, Sichuan University, Chengdu, Sichuan China; 2 Department of Epidemiology, Erasmus Medical Center, Rotterdam, The Netherlands; 3 Key Laboratory of Environment and Health, Ministry of Education & Ministry of Environmental Protection, and State Key Laboratory of Environmental Health, School of Public Health, Tongji Medical College, Huazhong University of Science and Technology, Wuhan, Hubei China; 4 Sichuan Health Information Center, Chengdu, Sichuan China; 5 School of Public Health, Kunming Medical University, Kunming, Yunnan China; 6 Department of Infectious Disease Epidemiology, London School of Hygiene and Tropical Medicine, London, UK

**Keywords:** Maternal care, Childhood immunization, Ethnicity

## Abstract

**Background:**

China has made remarkable progress in maternal and child health (MCH) over the last thirty years, but socio-economic inequalities persist. Ethnicity has become an important determinant of poor MCH outcomes, but little rigorous analytical work has been done in this area. To understand the socio-economic factors that explain ethnic variation in uptake of MCH care, we report the findings from an analysis in Sichuan province.

**Methods:**

We linked data from the 2003, 2008 and 2013 National Health Service Surveys in Sichuan Province. The ethnic disparities in uptake of maternal care (completing 5 antenatal visits, giving birth in hospital and receiving a caesarean section) and childhood immunization (Bacillus Calmette Guerin (BCG), three doses of diphtheria (DPT) and measles immunization) were examined by geographical (Han district/county vs. ethnic minority county) and individual-based (Han women/children vs. ethnic minority women/children) comparisons. We also examined variation by distance to township and county hospitals, women’s education, parity and age using weighted multilevel Poisson regressions with random intercept at district/county level.

**Results:**

Ethnic inequalities in maternal care were marked, both at the geographical (district/county) and the individual level. The % of births in hospital was 90.7% among women in Han districts, compared to 83.3% among women living in Han counties (crude RR 0.93; 95% CI 0.75–1.15), 53.8% among Han women living in ethnic minority counties (crude RR 0.57; 95% CI 0.36–0.93), and 13.5% among ethnic minority women living in ethnic minority counties (crude RR 0.18; 95% CI 0.06–0.57). Adjusting the analysis for survey year, education, parity and distance to county level hospital weakened the association between geographical/individual ethnicity and uptake of maternity care, but associations remained remarkably strong. Coverage of childhood immunization was much higher than uptake of maternity care, and inequalities by ethnicity were much less pronounced.

**Conclusion:**

Lessons can be learned from China’s successful immunization programme to further reduce inequalities in access to maternity care among ethnic minority populations in remote areas. Bringing the services closer to the women’s homes and strengthening health promotion from the township to the village level may encourage more women to seek antenatal care and give birth in hospital.

**Electronic supplementary material:**

The online version of this article (10.1186/s12884-019-2371-y) contains supplementary material, which is available to authorized users.

## Background

China has made remarkable progress in maternal and child health (MCH) over the last thirty years. The country achieved Millennium Development Goals 4 and 5 well before the target date [1, 2]. China’s success has been the result of committed efforts across all dimensions of the health system, including the provision of financial protection for women and children, investments in human resources for MCH, the introduction of evidence-based guidelines and the establishment of strong governance structures [3–7]. Beyond the health system, China’s substantial investments in education and infrastructure have greatly facilitated access to care [6].

Yet, inequalities in MCH care persist. A recent analysis in poor counties of Western China suggested that educational differences in access to antenatal and delivery care remained surprisingly large [2]. In a survey conducted in 2011, fewer than half of illiterate women in poor counties had given birth in health facilities, compared with nearly all women with college or higher education [2]. Studies have also shown markedly lower uptake of immunisation with BCG, DPT3, and measles among the poor and uneducated [8–10], though some studies have shown no inequalities by level of education [2].

In recent years, ethnicity has come to the forefront as an important determinant of poor MCH outcomes in China [11–13]. In a large national study reporting data for 1996 and 2012, children living in some ethnic minority counties had much higher infant mortality rates than children living in majority Han counties [12, 14]. In a study examining variation in maternal mortality between 1997 and 2014, the ethnic composition of the province was an important independent determinant of maternal mortality [2]. Apart from ecological comparisons where the geographically defined groups might be too diverse in ethnic composition to make meaningful comparisons, a recent systematic review also reported individual ethnic groups in China perform far worse than their Han counterparts in MCH outcomes and service coverage [15]. China’s 55 ethnic minorities are by no means homogeneous, however, and they represent diverse socioeconomic positions, languages, religions, and cultural and geographic contexts [16, 17]. Nearly three quarters (71.4%) of all ethnic minorities in China live in the Western Region [17], some of whom reside in remote mountainous areas with difficult access to care [18, 19]. Others, such as the Manchu or the Hui are much more urbanized, and their health status may not be that different from that of the Han [12].

There is remarkably little analytical work on the underlying reasons for health gaps between Han and ethnic minority populations in China. Ethnic minority status is generally thought to be associated with economic and educational disadvantages, rural residence, mountainous topography, poor infrastructure, and specific cultural norms, but empirical evidence on which factors are most important is lacking [13, 20]. Official Government reports tend to compare geographical units, e.g. Han and ethnic minority counties or prefectures, even though the ethnic composition of these units varies substantially [21]. Where comparisons are made between individuals from different ethnic backgrounds, attempts at explaining the ethnic variation are rarely made [13, 20].

The aim of this paper is to understand the socio-demographic factors that explain ethnic variation in uptake of MCH care in Sichuan province. Pooling data from three population-based surveys in 2003, 2008 and 2013, we report ethnic variation in uptake of antenatal care, birth in hospital, caesarean section and childhood immunization. Sichuan province is home to about 5 million ethnic minority people [16], the majority of whom belong to the Yi (2.6 million), Tibetan (1.5 million) and Qiang (0.3 million) ethnic groups. In 2014, 67 of the 183 districts/counties in Sichuan province were designated as ethnic minority counties [22]. By separating ethnicity from educational achievements, household wealth and distance to health facilities we aim to understand which factors contribute to poor uptake of MCH care among ethnic minorities in Sichuan province.

## Methods

We used data from three cross-sectional Health Service Surveys conducted in Sichuan province in 2003, 2008 and 2013. These surveys are part of the National Health Service Surveys which have been carried out in China every five years since 1993 [23]. All surveys stratified the population by urban and rural areas, using the administrative subdivisions of district, street and community for urban areas; and county, town and village for rural areas. The 2003 and 2008 surveys used three-stage stratified cluster random sampling to select households. In the first stage, the province was stratified by streets and towns and 30 streets/towns were randomly sampled within each stratum. In the second stage two communities/villages were randomly sampled within each street/town, and in the third stage a systematic sample of 33 households was selected from each community/village [24]. The 2013 survey used a different sampling strategy and was based on four-stage stratified random sampling [25]. In the first stage, districts and counties were stratified and seven districts/counties were randomly sampled within each stratum. In the second stage five streets/towns were sampled systematically from selected districts/counties; in the third stage two communities/villages were sampled randomly from selected streets/towns and in the fourth stage 60 households were sampled systematically within each community/village.

All surveys used locally trained doctors or public health professionals from community health centers/township hospitals or county level health bureaus to interview households using a structured questionnaire. One section in the questionnaire provided information on characteristics of births occurring within five years preceding the survey and another section provided information on children aged under five at the time of the survey. All surveys recorded information for the last live birth only, enquiring where the birth had taken place, the number of antenatal visits, and the mode of delivery. For children aged one to four years old, mothers or caregivers were asked whether the child had received Bacillus Calmette Guerin (BCG) immunization, the number of doses of diphtheria, tetanus and pertussis (DPT) immunization, and whether the child had been immunized for measles (1 dose).

**Fig. 1:**
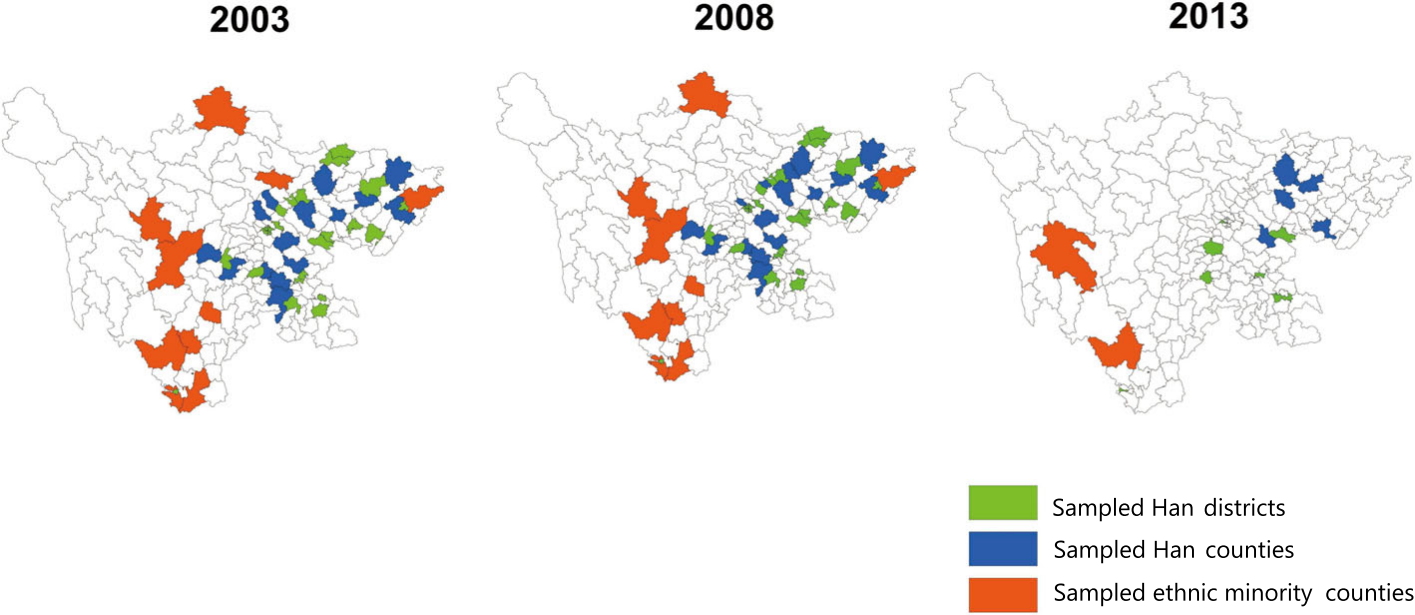
Geographical distribution of 2003, 2008 and 2013 National Health Service Surveys sampled counties in Sichuan Province

Ethnicity was our main exposure variable. We defined ethnicity by geographical and individual ethnicity. First, districts (referring to urban communities) and counties (referring to rural communities) were labeled as Han or ethnic minority based on the Chinese Government’s categorization of whether the district/county qualified as an ethnic minority area [22]. The Government categorizes districts and counties as ethnic minority mainly depending on the proportion of ethnic minority people living there [17]. The 2003 and 2008 survey sampled 21 Han districts, 20 Han counties, and 10 and 9 ethnic minority counties respectively (Fig. 1). Corresponding figures for the 2013 survey were 7, 5 and 2 respectively. The predominant ethnicity in the sampled ethnic minority counties was Tibetan (three counties in 2003/2008 and one in 2013), Yi (six counties in 2003/2008 and one in 2013) and Qiang (one county in 2003). Second, individuals living within ethnic minority counties were further classified as being Han or ethnic minority, based on individuals’ reports at the time of the survey. The questionnaires recorded whether individuals belonged to Tibetan, Meng, Hui, and Miao ethnic groups, but the “other” ethnic groups were not specified (see Additional file 1).

We selected the following potential confounders for the association between ethnicity and uptake of MCH care: household’s annual income, mother’s educational attainment, maternal age, parity and distance to health facilities. Per capita income was obtained by dividing annual household income divided by household size. The quartiles were used as the measures of per capita income. Mother’s educational attainment were categorized into four groups: no formal schooling, completed primary, completed secondary and college and above. In 2003 and 2008, obstetrics services were provided in primary care centers (township hospitals and community health centers) as well as county/district and higher level hospitals (including MCH hospitals); immunization services were mostly provided at the primary care level. In 2013, many primary care centers stopped providing obstetric care. We computed two measures of distance to health facilities. First, the distance to community/township level hospitals measures the straight-line distance between the geographic center of the street/village to the nearest community health center/township hospital. Second, distance to hospital at district/county level measures the straight-line distance between the geographic center of the street/village to the nearest district/county hospital. All calculations were based on the latitude and longitude of the corresponding hospitals or streets/villages geo-located by google earth [26].

Our analysis was restricted to live births and children aged one to four, depending on the indicator used in the surveys. For live births, we report the proportion of births with five or more antenatal visits (according to the National Basic Public Health Service Standards 2011 [27]), in hospitals (including community health centers/township hospitals, MCH facilities, county/district hospitals and above), and by caesarean section. For children aged one to four we report the proportion who received at least one dose of BCG, measles vaccination and 3 doses of DPT. All immunization data were based on the mother’s or caregiver’s report. To make our findings representative of Sichuan province, data were weighted at district/county and individual levels. We calculated the district/county level weight by dividing the total number of districts/counties by the sampled number of districts/counties. We calculated the individual level weight by dividing the population size of selected districts/counties by the sampled population size of selected districts/counties. The population size of each district/county was obtained from the Statistical Yearbooks of Sichuan province [28–30].

We pooled the data from the three surveys, used multilevel weighted Poisson regression models with random intercept at district/county level assuming that individual woman and child (lower hierarchy) and the district/county in where they live (higher hierarchy) share similar background characteristics and are therefore clustered [31]. We estimated crude and adjusted risk ratios for the six outcomes across socio-demographic characteristics. We used the models to conduct variables selection. Per capita income, women’s age, parity, and distance to community health center/township hospital were therefore included when the univariate test was significant at the 0.05 level and it has explained the estimated district/county level differences by at least 10% compared to the null model. Year of survey, maternal education and distance to the nearest hospital were considered the priori confounders for the association between ethnicity and all outcomes, and were therefore included in all adjusted models. Other variables were included if the model converged. All analyses were done using the gllamm package in STATA (version 13) and the statistical software R (Version 3.1).

## Results

### Characteristics of the sample

The sample consisted of 1754 women with a birth in the last five years, and 1820 children aged one to four at the time of the survey. For the analysis of data from children aged one to four, maternal education, age and parity were missing for 492 (27.0%) children because the mothers were not present at the time of the survey. These children were retained in the analysis and categories labelled missing were displayed separately.

Nearly half of all women (45.8%) lived in Han districts, 22.7% lived in Han counties, and 31.5% lived in ethnic minority counties. A tiny fraction of those living in Han districts (3.4, 3.8, and 2.8% in 2003, 2008 and 2013 respectively) or Han counties (1.0, 0.0, and 0.5% in 2003, 2008 and 2013 respectively) belonged to ethnic minorities. Among those living in ethnic minority counties, the large majority (84.6, 85.7, and 85.6% in 2003, 2008 and 2013 respectively) were ethnic minority women.

Women living in ethnic minority counties were much poorer and less educated than women living in Han districts/counties (Table 1). For example, only 3.2 and 5.1% respectively of those living in Han districts/counties had no formal education, compared to 14.8% of Han and 72.0% of ethnic minority women living in ethnic minority counties. Women living in ethnic minority counties were also much more likely to live far from a county hospital. Only 1.6 and 15.9% respectively of those living in Han districts/counties lived more than 30 km away from a district/county level hospital compared to 33.3% of Han and 72.2% of ethnic minority women from ethnic minority counties. The socio-demographic characteristics of children aged one to four were similar to those of the women (Table 2).

**Table 1:** Socio-demographic characteristics of women in Sichuan Province (2003, 2008, 2013 National Health Service Surveys)

		Ethnicity and place of residence				*P*-value*
		Han districts N (%)	Han counties N (%)	Ethnic minority counties		
				Han women N (%)	Ethnic minority women N (%)	
Year	2003	265 (32.9)	101 (25.4)	25 (30.9)	137 (29.1)	0.0161
	2008	212 (26.4)	100 (25.1)	17 (21.0)	102 (21.6)	
	2013	327 (40.7)	197 (49.5)	39 (48.1)	232 (49.3)	
Mother’s education	None	26 (3.2)	20 (5.1)	12 (14.8)	339 (72.0)	<0.0001
	Completed primary	114 (14.2)	104 (26.1)	30 (37.0)	104 (22.1)	
	Completed secondary	427 (53.1)	250 (62.8)	34 (42.0)	25 (5.3)	
	College and above	237 (29.5)	24 (6.0)	5 (6.2)	2 (0.4)	
	Missing	-	-	-	1 (0.2)	
Per capita income	1^st^ quartile	90 (11.1)	75 (18.8)	26 (32.1)	274 (58.2)	<0.0001
	2^nd^ quartile	141 (17.5)	116 (29.2)	25 (30.9)	141 (29.9)	
	3^rd^ quartile	236 (29.4)	133 (33.4)	20 (24.7)	46 (9.8)	
	4^th^ quartile	337 (41.9)	74 (18.6)	10 (12.3)	10 (2.1)	
Mother’s age	15-24	155 (19.3)	96 (24.1)	29 (35.8)	137 (29.1)	<0.0001
	25-34	535 (66.5)	217 (54.5)	47 (58.0)	245 (52.0)	
	>=35	114 (14.2)	85 (21.4)	5 (6.2)	89 (18.9)	
Parity	1	592 (73.7)	192 (48.2)	34 (42.0)	130 (27.6)	< 0.0001
	2	186 (23.1)	194 (48.8)	36 (44.4)	174 (36.9)	
	>=3	25 (3.1)	12 (3.0)	11 (13.6)	167 (35.5)	
	Missing	1 (0.1)	-	-	-	
Distance to community/ township level hospital (km)	<1	309 (38.4)	105 (26.4)	46 (56.8)	109 (23.1)	< 0.0001
	1.0-1.9	221 (27.5)	99 (24.9)	8 (9.9)	33 (7.0)	
	2.0-4.9	201 (25.0)	125 (31.4)	9 (11.1)	96 (20.4)	
	>=5.0	73 (9.1)	69 (17.3)	18 (22.2)	233 (49.5)	
Distance to district/county level hospital (km)	<3.0	330 (41.0)	63 (15.8)	16 (19.8)	21 (4.4)	< 0.0001
	3.0-8.9	270 (33.6)	90 (22.6)	15 (18.5)	24 (5.1)	
	9.0-29.0	191 (23.8)	182 (45.7)	23 (28.4)	86 (18.3)	
	>=30.0	13 (1.6)	63 (15.9)	27 (33.3)	340 (72.2)	
All		804 (100.0)	398 (100.0)	81 (100.0)	471 (100.0)	

*all hypothesis tests used Chi-square test

**Table 2:** Socio-demographic characteristics of children aged 1 to 4 in Sichuan Province (2003, 2008, 2013 National Health Service Surveys)

		Ethnicity and place of residence				*P*-value*
		Han districts N (%)	Han counties N (%)	Ethnic minority counties		
				Han children N (%)	Ethnic minority children N (%)	
Year	2003	191 (24.3)	73 (14.9)	10 (14.3)	101 (21.2)	<0.0001
	2008	193 (24.6)	130 (26.6)	15 (21.4)	105 (22.1)	
	2013	401 (51.1)	286 (58.5)	45 (64.3)	270 (56.7)	
Mother’s education	None	22 (2.8)	14 (2.9)	12 (17.2)	316 (66.4)	<0.0001
	Completed primary	89 (11.3)	84 (17.2)	24 (34.3)	92 (19.3)	
	Completed secondary	280 (35.7)	167 (34.1)	19 (27.1)	23 (4.9)	
	College and above	163 (20.8)	16 (3.3)	4 (5.7)	3 (0.6)	
	Missing	231 (29.4)	208 (42.5)	11 (15.7)	42 (8.8)	
Per capita income	1^st^ quartile	114 (14.5)	90 (18.4)	26 (37.1)	268 (56.3)	<0.0001
	2^nd^ quartile	153 (19.5)	132 (27.0)	15 (21.4)	154 (32.4)	
	3^rd^ quartile	212 (27.0)	156 (31.9)	20 (28.6)	42 (8.8)	
	4^th^ quartile	306 (39.0)	111 (22.7)	9 (12.9)	12 (2.5)	
Mother’s age	15-24	74 (9.4)	55 (11.3)	18 (25.7)	113 (23.7)	<0.0001
	25-34	389 (49.6)	154 (31.5)	37 (52.9)	245 (51.5)	
	>=35	91 (11.6)	72 (14.7)	4 (5.7)	76 (16.0)	
	Missing	231 (29.4)	208 (42.5)	11 (15.7)	42 (8.8)	
Parity	1	384 (48.9)	119 (24.3)	16 (22.9)	93 (19.6)	<0.0001
	2	150 (19.1)	153 (31.3)	35 (50.0)	181 (38.0)	
	>=3	20 (2.6)	9 (1.9)	8 (11.4)	160 (33.6)	
	Missing	231 (29.4)	208 (42.5)	11 (15.7)	42 (8.8)	
Distance to community/ township level hospital(km)	<1	265 (33.7)	120 (24.4)	42 (60.0)	116 (24.4)	<0.0001
	1.0-1.9	203 (25.9)	123 (25.2)	8 (11.4)	32 (6.7)	
	2.0-4.9	229 (29.2)	167 (34.2)	13 (18.6)	97 (20.4)	
	>=5.0	88 (11.2)	79 (16.2)	7 (10.0)	231 (48.5)	
Distance to district/county level hospital (km)	<3.0	300 (38.2)	65 (13.3)	15 (21.4)	22 (4.6)	<0.0001
	3.0-8.9	218 (27.8)	111 (22.7)	11 (15.7)	23 (4.8)	
	9.0-29.0	239 (30.4)	242 (49.5)	13 (18.6)	75 (15.8)	
	>=30.0	28 (3.6)	71 (14.5)	31 (44.3)	356 (74.8)	
All		785 (100.0)	489 (100.0)	70 (100.0)	476 (100.0)	

*all hypothesis tests used Chi-square test

### Uptake of maternity care

**Fig. 2:**
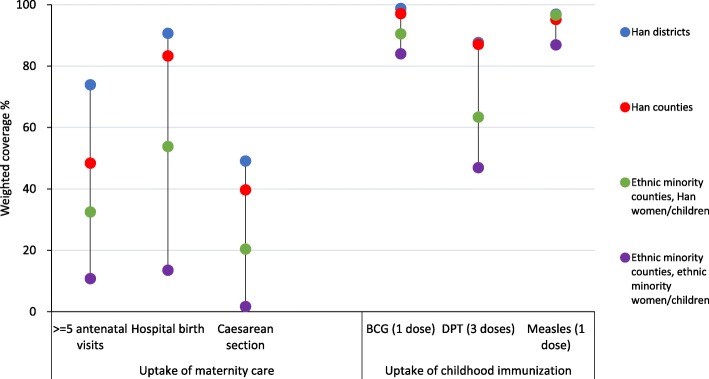
Uptake of antenatal care, delivery care, caesarean section and childhood immunization of women and children in Sichuan province by geographical/individual ethnicity (2003, 2008, 2013 National Health Service Surveys)

Geographical ethnicity and individual ethnicity were strongly associated with all three indicators of uptake of maternity care (Table 3, Fig. 2). Ethnic minority women living in ethnic minority counties had extremely low uptake of antenatal care (10.8%), facility delivery (13.5%) and caesarean section (1.7%) (Fig. 2). For all three indicators of maternity care uptake was lowest among ethnic minority women living in ethnic minority counties, increasing progressively among Han women living in ethnic minority counties, women living in Han counties and Han districts. For example, the % of births in hospital was 90.7% among women in Han districts, compared to 83.3% among women living in Han counties (crude RR 0.93; 95% CI 0.75–1.15), 53.8% among Han women living in ethnic minority counties (crude RR 0.57; 95% CI 0.36–0.93), and 13.5% among ethnic minority women living in ethnic minority counties (crude RR 0.18; 95% CI 0.06–0.57).

**Table 3:** Uptake of antenatal care, delivery care and caesarean sections of women in Sichuan Province (2003, 2008, 2013 National Health Service Surveys)

			N	Antenatal care (>= 5 visits)			Hospital birth			Caesarean section		
				%^a^	Unadjusted RR (95% CI ^b^)	Adjusted^d^ RR (95% CI^b^)	% ^a^	Unadjusted RR (95% CI ^b^)	Adjusted ^d^ RR (95% CI ^b^)	% ^a^	Unadjusted RR (95% CI ^b^)	Adjusted ^d^ RR (95% CI ^b^)
Ethnicity and place of residence	Han districts		804	73.9	1.00	1.00	90.7	1.00	1.00	49.1	1.00	1.00
	Han counties		398	48.4	0.75^*^ (0.57-0.99)	0.82 (0.66-1.03)	83.3	0.93 (0.75-1.15)	1.05 (0.93-1.20)	39.7	0.73^*^ (0.54-0.98)	0.92 (0.72-1.16)
	Ethnic minority counties	Han women	81	32.5	0.60 (0.29-1.24)	0.66 (0.35-1.25)	53.8	0.57^*^ (0.36-0.93)	0.79 (0.62-1.00)	20.4	0.39^*^ (0.20-0.77)	0.57^*^ (0.36-0.90)
	Ethnic minority counties	Ethnic minority women	471	10.8	0.13^*^ (0.09-0.18)	0.22^*^ (0.10-0.49)	13.5	0.18^*^ (0.06-0.57)	0.45^*^ (0.27-0.70)	1.7	0.04^*^ (0.02-0.10)	0.12^*^ (0.06-0.25)
Year	2003		528	30.0	1.00	1.00	42.1	1.00	1.00	11.3	1.00	1.00
	2008		431	33.2	1.02 (0.70-1.48)	0.99 (0.70-1.38)	61.3	1.45^*^ (1.18-1.77)	1.36^*^ (1.12-1.60)	28.8	2.55^*^ (1.85-3.50)	2.33^*^ (1.72-3.16)
	2013		795	44.8	1.75^*^ (1.17-2.61)	1.26 (0.89-1.79)	60.1	1.40 ^*^ (1.09-1.80)	1.32^*^ (1.08-1.60)	31.8	3.06^*^ (1.63-5.74)	2.37^*^ (1.79-3.14)
Women’s education^c^	None		397	9.5	1.00	1.00	9.7	1.00	1.00	1.7	1.00	1.00
	Completed primary		352	24.5	2.13^*^ (1.45-3.12)	1.57^*^ (1.17-2.12)	50.3	3.42^*^ (1.85-6.31)	2.5^*^ (1.56-4.00)	15.6	6.45^*^ (1.7-24.44)	2.55^*^ (0.82-7.89)
	Completed secondary		736	59.0	3.88^*^ (2.78-5.42)	2.35^*^ (1.6-3.46)	87.8	4.52^*^ (2.04-10.05)	2.83^*^ (1.71-4.70)	44.2	13.14^*^ (3.75-46.09)	3.98^*^ (1.42-11.19)
	College and above		268	86.0	4.87^*^ (3.48-6.80)	2.55^*^ (1.74-3.73)	97.8	4.82^*^ (2.09-11.15)	2.83^*^ (1.72-4.70)	57.7	15.34^*^ (4.37-53.94)	4.24^*^ (1.49-12.07)
	Missing		1	-	-	-	-	-	-	-	-	-
Per capita income^e^	1^st^ quartile		465	12.7	1.00	-	26.7	1.00	-	9.7	1.00	-
	2^nd^ quartile		423	35.3	2.01^*^ (1.22-3.29)	-	47.7	1.25^*^ (1.06-1.49)	-	20.8	1.35 (1.00-1.83)	-
	3^rd^ quartile		435	54.4	2.11^*^ (1.43-3.13)	-	78.9	1.37^*^ (1.07-1.75)	-	34.6	1.34 (0.97-1.84)	-
	4^th^ quartile		431	70.8	2.22^*^ (1.51-3.26)	-	94.9	1.43^*^ (1.11-1.86)	-	52.9	1.60^*^ (1.13-2.28)	-
Women’s age^e^	15-24		417	38.0	1.00	-	53.9	1.00	-	22.1	1.00	-
	25-34		1044	40.4	0.88 (0.76-1.03)	-	55.5	0.89^*^ (0.83-0.95)	-	24.8	0.94 (0.82-1.08)	-
	>=35		293	30.1	0.66^*^ (0.49-0.9)	-	57.9	0.91^*^ (0.84-0.99)	-	32.6	1.16 (0.94-1.43)	-
Parity^c^	1		948	54.5	1.00	1.00	73.9	1.00	1.00	35.1	1.00	-
	2		590	30.8	0.73^*^ (0.63-0.84)	0.80^*^ (0.70-0.91)	51.9	0.88^*^ (0.79-0.97)	0.89 (0.81-1.00)	23.0	0.89 (0.76-1.04)	-
	>=3		215	9.3	0.42^*^ (0.27-0.65)	0.54^*^ (0.41-0.72)	14.0	0.49^*^ (0.32-0.75)	0.64 (0.49-0.8)	5.4	0.61 (0.36-1.04)	-
	Missing		1	-	-	-	-	-	-	-	-	-
Distance to community/township level hospital (km)	<1.0		569	46.4	1.00	-	64.9	1.00	-	34.1	1.00	-
	1.0-1.9		361	62.6	1.31 (0.86-1.99)	-	78.3	0.94 (0.83-1.06)	-	38.8	1.22 (0.90-1.66)	-
	2.0-4.9		431	30.3	0.86 (0.63-1.19)	-	60.4	1.00 (0.82-1.21)	-	23.2	0.92 (0.67-1.27)	-
	>=5.0		393	20.8	0.83 (0.56-1.23)	-	27.7	0.68^*^ (0.49-0.94)	-	10.6	0.58^*^ (0.37-0.91)	-
Distance to district/county level hospital (km)	<3.0		430	72.8	1.00	1.00	91.7	1.00	1.00	54.6	1.00	1.00
	3.0-8.9		399	53.7	0.87 (0.71-1.08)	0.93 (0.77-1.14)	86.5	0.94 (0.84-1.04)	1.00 (0.88-1.20)	37.6	0.86 (0.67-1.11)	0.80 (0.64-1.00)
	9.0-29.0		482	34.8	0.69^*^ (0.57-0.84)	0.84 (0.68-1.04)	64.0	0.85 (0.73-1.00)	0.87 (0.75-1.00)	26.4	0.70^*^ (0.55-0.88)	0.72^*^ (0.59-0.87)
	>=30.0		443	16.4	0.58 (0.27-1.27)	0.91 (0.57-1.46)	16.6	0.33^*^ (0.20-0.54)	0.55^*^ (0.34-0.90)	5.2	0.19^*^ (0.12-0.29)	0.51^*^ (0.27-0.98)
All			1754	59.9	-	-	76.6	-	-	36.4	-	-

^a^all proportion displayed were weighted by county and individual

^b^confidence interval

^c^mothers’ age and parity values were missing for 1 woman

^d^adjusted variables: Antenatal care (>= 5 visits): year, women’s education, parity, distance to district/county level hospital (km); Hospital birth: year, women’s education, parity, distance to district/county level hospital (km); Caesarean section: year, women’s education, distance to district/county level hospital (km)

^e^adjusted model did not converge for including the variable

^*^P<0.05

Uptake of antenatal care increased substantially between 2008 and 2013 (Table 3). In contrast, the proportion of births in hospital increased from 42.1% in 2003 to 61.3% in 2008 (crude RR 1.45; 95% CI 1.18–1.77), but remained largely unchanged at 60.1% in 2013 (crude RR 1.40; 95% CI 1.09–1.80). Similarly, caesarean sections increased dramatically between 2003 and 2008, leveling out at a third of all births (31.8%) in 2013. Among women giving birth in hospital most gave birth in county or higher level hospitals (including MCH hospitals), with little change over time (73.2, 76.1 and 84.5% of all births in 2003, 2008 and 2013 survey respectively). Community health center/township hospitals only accounted for 26.8, 23.9 and 15.5% of hospital-based births in 2003, 2008, and 2013 respectively (see Additional file 2).

Education, income and distance to the district/county level hospital were strongly associated with all maternal care indicators in the crude analyses. Among women living more than 30 km from a district/county level hospital, for example, only 16.6% delivered in a health facility, compared to 91.7% for women living within 3 km (crude RR 0.33; 95% CI 0.20–0.54).

Adjusting the analysis for survey year, education, parity and distance to district/county level hospital weakened the association between geographical/individual ethnicity and uptake of maternity care, but the association remained remarkably strong (Table 3). For example, after taking account of socio-demographic factors, ethnic minority women remained four times less likely than urban Han women to have five or more antenatal visits (adjusted RR 0.22; 95% CI 0.10–0.49), two times less likely to give birth in hospital (adjusted RR 0.45; 95% CI 0.27–0.70) and eight times less likely to have a caesarean section (adjusted RR 0.12; 95% CI 0.06–0.25). Women’s education and distance to the district/county level hospital remained important predictors of birth in hospital or caesarean section, even after adjusting for other socio-economic variables (Table 3).

**Fig. 3:**
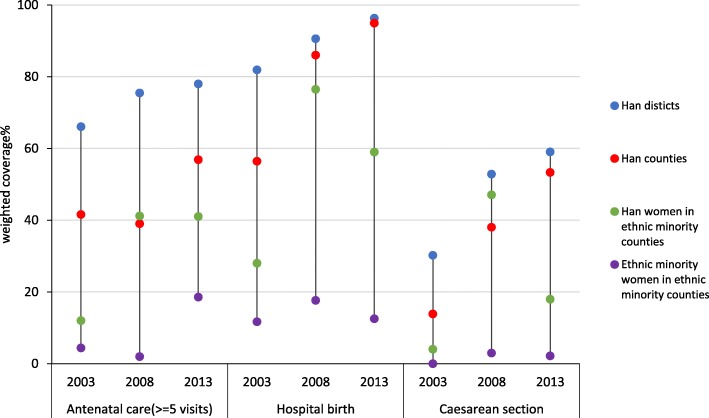
Uptake of antenatal care, delivery care and caesarean section of women in Sichuan province by geographical/individual ethnicity and survey year (2003, 2008, 2013 National Health Service Surveys)

Figure 3 shows changes over time in uptake of maternity care by geographical and individual ethnicity. Inequalities in access to maternity care persisted over time, and there was no evidence of progress among ethnic minority women living in ethnic minority areas. We repeated the Poisson model with an interaction term for survey year and geographical/individual ethnicity, but the model did not converge.

### Childhood immunization coverage

**Fig. 4:**
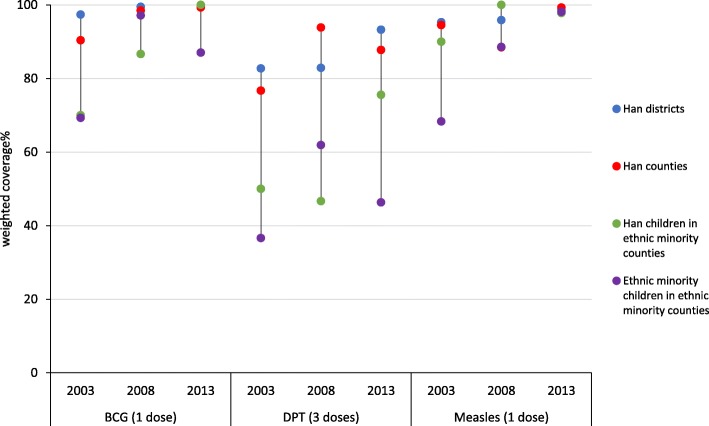
Uptake of childhood immunization of children aged 1 to 4 in Sichuan province by geographical/individual ethnicity and survey year (2003, 2008, 2013 National Health Service Surveys)

Childhood immunization coverage was much higher than uptake of maternity care, increasing over time for all vaccines (Table 4). BCG and measles immunization were high regardless of geographical/individual ethnicity, though ethnic minority children in ethnic minority counties had somewhat lower uptake of BCG than urban children (84%, crude RR 0.88; 95% CI 0.82–0.94) (Table 4, Fig. 4). Coverage of three doses of DPT was lower among children living in ethnic minority counties compared to urban children, whether they were Han (63.4%, RR 0.79; 95% CI 0.64–0.98) or belonged to ethnic minorities (46.9%, crude RR 0.63; 95% CI 0.44–0.90). Mother’s education, parity, household income and distance to community/township level hospital were associated with BCG and DPT immunization. For example, only 47.5% of children from mothers with no formal education received three doses of DPT, compared to 85.7% of children from mothers with college or higher education (crude RR 1.45; 95% CI 1.09–1.93). Adjusting the analysis for survey year, education, household income and distance to community/township level hospital weakened the association between ethnicity and uptake of childhood immunization, but the lower coverage of BCG and DPT among ethnic minority children in ethnic minority counties persisted (Table 4).

**Table 4:** Uptake of childhood immunization of children aged 1 to 4 in Sichuan Province (2003, 2008, 2013 National Health Service Surveys)

			N	BCG immunization (1 dose)			DPT immunization (3 doses)			Measles immunization (1 dose)		
				% ^a^	Unadjusted RR (95% CI)	Adjusted ^c^ RR (95% CI)	% ^a^	Unadjusted RR (95% CI)	Adjusted ^c^ RR (95% CI)	% ^a^	Unadjusted RR (95% CI)	Adjusted ^c^ RR (95% CI)
Ethnicity and place of residence	Han districts		785	98.8	1.00	1.00	87.7	1.00	1.00	96.9	1.00	1.00
	Han counties		489	97.1	0.99 (0.97-1.01)	0.98 (0.95-1.01)	87.1	1.00 (0.90-1.12)	1.00 (0.91-1.09)	95.2	0.98 (0.94-1.03)	0.98 (0.94-1.02)
	Ethnic minority counties	Han children	70	90.5	0.99 (0.98-1.01)	1.03 (1.00-1.06)	63.4	0.76^*^ (0.64-0.90)	0.79^*^ (0.64-0.98)	96.6	1.00 (0.98-1.03)	1.01 (0.94-1.08)
	Ethnic minority counties	Ethnic minority children	476	84.0	0.88^*^ (0.82-0.94)	0.93^*^ (0.87-0.99)	46.9	0.56^*^ (0.37-0.85)	0.63^*^ (0.44-0.90)	86.9	0.91 (0.81-1.03)	0.93 (0.83-1.03)
Year	2003		375	82.3	1.00	1.00	60.1	1.00	1.00	83.5	1	1.00
	2008		443	97.6	1.23^*^ (1.06-1.42)	1.23^*^ (1.06-1.43)	78.5	1.31^*^ (1.04-1.64)	1.28^*^ (1.02-1.60)	90.4	1.07 (0.93-1.23)	1.08 (0.94-1.24)
	2013		1002	94.9	1.14 (0.98-1.33)	1.14 (0.99-1.32)	73.0	1.41 (1.00-1.97)	1.20 (0.95-1.52)	98.6	1.17^*^ (1.03-1.33)	1.18^*^ (1.02-1.35)
Mother’s education	None		364	82.1	1.00	1.00	47.5	1.00	1.00	87.6	1.00	1.00
	Completed primary		289	88.9	1.08 (1.00-1.16)	1.04 (0.97-1.11)	66.5	1.27^*^ (1.05-1.55)	1.06 (0.88-1.27)	88.2	1.00 (0.90-1.10)	0.97 (0.89-1.06)
	Completed secondary		489	99.4	1.16^*^ (1.08-1.25)	1.10^*^ (1.04-1.16)	85.6	1.47^*^ (1.16-1.87)	1.10 (0.90-1.35)	97.0	1.09 (0.98-1.20)	1.03 (0.96-1.10)
	College and above		186	99.6	1.16^*^ (1.08-1.25)	1.09^*^ (1.04-1.15)	85.7	1.45^*^ (1.09-1.93)	1.06 (0.84-1.35)	97.1	1.08 (0.98-1.21)	1.02 (0.96-1.09)
	Missing		492	97.8	1.16^*^ (1.08-1.24)	1.04 (1.00-1.09)	83.9	1.47^*^ (1.16-1.86)	1.04 (0.85-1.27)	96.3	1.08 (0.96-1.20)	0.98 (0.91-1.06)
Per capita income	1^st^ quartile		498	87.4	1.00	1.00	57.7	1.00	1.00	90.6	1.00	-
	2^nd^ quartile		454	89.3	1.00 (0.95-1.06)	0.98 (0.94-1.02)	65.5	1.10 (0.94-1.28)	1.01 (0.87-1.17)	90.6	1.00 (0.95-1.05)	-
	3^rd^ quartile		430	98.0	1.07^*^ (1.02-1.14)	1.01 (0.96-1.07)	84.0	1.23^*^ (1.08-1.41)	1.10 (0.97-1.25)	94.6	1.03 (0.96-1.10)	-
	4^th^ quartile		438	98.6	1.08^*^ (1.03-1.13)	1.01 (0.96-1.06)	87.8	1.25^*^ (1.12-1.39)	1.10 (0.99-1.22)	96.6	1.05 (0.99-1.12)	-
Mother’s age ^b^	15-24		260	88.7	1.00	-	62.9	1.00	-	89.8	1.00	-
	25-34		825	91.6	1.00 (0.96-1.05)	-	68.2	1.00 (0.91-1.09)	-	92.2	1.02 (0.97-1.07)	-
	>=35		243	88.7	0.97 (0.9-1.05)	-	67.6	1.02 (0.96-1.09)	-	90.6	1.01 (0.94-1.08)	-
	Missing		492	97.8	1.07^*^ (1.01-1.13)	-	83.9	1.11 (0.97-1.26)		96.3	1.05 (0.96-1.15)	-
Parity ^b^	1		612	95.0	1.00	-	78.5	1.00	-	91.9	1.00	-
	2		519	90.5	0.97 (0.94-1.01)	-	67.1	0.93 (0.87-1.00)	-	91.4	0.99 (0.96-1.03)	-
	>=3		197	80.8	0.88^*^ (0.82-0.95)	-	42.5	0.72^*^ (0.63-0.83)	-	90.2	1.00 (0.92-1.09)	-
	Missing		492	97.8	1.04 (0.99-1.08)	-	83.9	1.04 (0.96-1.14)	-	96.3	1.04 (0.97-1.11)	-
Distance to community/township level hospital (km)	<1		543	88.8	1.00	1.00	69.6	1.00	1.00	91.4	1.00	1.00
	1.0-1.9		366	97.7	1.09^*^ (1.01-1.18)	1.08^*^ (1.02-1.16)	84.8	1.09^*^ (1.01-1.18)	1.10 (0.94-1.28)	95.5	1.03 (0.93-1.13)	1.03 (0.95-1.13)
	2.0-4.9		506	96.5	1.08 (0.99-1.17)	1.09^*^ (1.01-1.19)	76.8	1.08 (0.99-1.17)	1.10 (0.91-1.32)	95.2	1.03 (0.95-1.11)	1.05 (0.97-1.13)
	>=5.0		405	87.8	0.99 (0.92-1.07)	1.07 (0.98-1.17)	57.6	0.99 (0.92-1.07)	1.06 (0.93-1.2)	89.0	0.97 (0.88-1.08)	1.03 (0.92-1.15)
Distance to district/county level hospital (km)	<3.0		402	99.7	1.00	-	84.0	1.00	-	97.8	1.00	-
	3.0-8.9		363	97.6	0.99 (0.96-1.01)	-	84.4	0.99 (0.96-1.01)	-	93.5	0.95 (0.89-1.02)	-
	9.0-29.0		569	92.8	0.95 (0.89-1.01)	-	80.1	0.95 (0.89-1.01)	-	90.4	0.93 (0.86-1.02)	-
	>=30.0		486	86.2	0.90^*^ (0.84-0.97)	-	51.3	0.90^*^ (0.84-0.97)	-	91.9	0.95 (0.89-1.01)	-
All			1820	96.8	-	-	80.3	-	-	94.3	-	-

^a^all proportion displayed were weighted by county and individual

^b^mothers’ age and parity values were missing for 492 children

^c^adjusted variables: BCG immunization (1 dose): year, mother’s education, per capita income, distance to community/township level hospital (km); DPT immunization (3 doses): year, mother’s education, per capita income, distance to community/township level hospital (km); Measles immunization (1 dose): year, mother’s education, distance to community/township level hospital (km)

^*^P<0.05

Figure 4 shows changes over time in uptake of childhood immunization by geographical and individual ethnicity. Inequalities in BCG and measles immunization reduced over time, while uptake of 3 doses of DPT remained much lower in children living in ethnic minority counties compared to those living in Han districts or counties. We also repeated the Poisson model with an interaction term for survey year and residence/ethnicity, but the model did not converge.

## Discussion

Our analysis of three consecutive surveys in Sichuan province found strong evidence of poorer uptake of antenatal care, delivery care, and caesarean section among ethnic minority women living in ethnic minority counties compared with Han women living in majority Han districts or counties. Han women living in ethnic minority counties also had lower uptake of maternity care compared to Han women living in Han districts or counties. Mother’s education, parity and distance to district/county level hospitals were strong independent predictors of uptake of maternity care, but ethnic differentials remained substantial even after taking account of these factors. Inequalities in childhood immunization were much smaller than those for maternity care, though coverage with 3 doses of DPT immunization was low among Han and ethnic minority children from ethnic minority counties.

Uptake of maternity care among ethnic minority women in this study was extremely low, particularly given the high uptake reported nationally in 2008 and 2013 [32, 33]. In the 2013 National Health Service Survey, 69.1% of Chinese women had received five or more antenatal care visits, 96.3% gave birth in hospital and 41.0% had a caesarean section [33]. In our survey, only 10.8% of ethnic minority women had received five or more antenatal visits, 13.5% gave birth in hospital, and 1.7% had a caesarean section. Equally low levels of uptake of maternity care have been reported previously for ethnic minority women living across Western China [15, 34–36]. The extremely low caesarean section rates are particularly worrying: while caesarean section rates cannot be a substitute for the measurement of levels of maternal mortality, research has found that the caesarean section rate is negatively associated with maternal mortality when under 10% [37], and rates below 5% suggest an unmet need for life saving surgery [38]. The ethnic minority women in ethnic minority counties in our study were mostly of the Yi and Tibetan ethnicity and nearly three quarters (72.0%) were uneducated, or lived more than 30 km away from the nearest county hospital (72.2%). Most of the Yi and Tibetans live in remote areas with levels of economic and social development that are far lower than the provincial and national average, and with transport infrastructure that is relatively undeveloped [30, 39, 40].

Childhood immunization coverage, in contrast, was higher, and coverage of BCG and measles immunization increased over time, particularly among ethnic minorities. The overall levels reported here were consistent with those reported nationally: in the 2013 survey national coverage was 98.7% for BCG, 92.5% for three doses of DPT and 97.3% for measles [33]. Even for ethnic minority children in our survey, the coverage of BCG and measles immunization (over 80%) was similar to what has been found in other Western provinces in China [15, 35]. Childhood immunization has been the National Government’s priority since the 1970s [41], and coverage has been generally high [42, 43]. Immunization in rural areas is mostly delivered at township level hospitals, close to the people’s homes, with dedicated staff working on fixed days of the month [44]. The programme is further supported by outreach to villages, through village doctors who offer health education and vaccination itself – provided they are certified to do so. Children from ethnic minority counties still had low uptake of three doses of DPT however, whether they were Han or ethnic minority. Coverage of BCG immunization we report here is high, but other studies have suggested that BCG is sometimes given much later than the first day after birth, and coverage of timely BCG may thus be much lower than what we report here [45].

Lower uptake of MCH care in ethnic minority counties, whether the women are Han or ethnic minority, suggest that wider environmental factors such as mountainous terrain and poor road infrastructure may be as important as individual or household characteristics in explaining the gaps in uptake of MCH care between Han and ethnic minority populations. The greater inequalities in maternity care compared to childhood immunization further suggest that specific service delivery models may be able to overcome these barriers, at least in part. Delivery care in rural areas in China is increasingly offered in large county hospitals [2, 6], while immunization is largely offered in township hospitals, which are closer to the women’s home [44]. Ethnic differentials in access to maternal care remained surprisingly large after adjusting for socio-demographic factors and distance to the nearest health facilities. Although there may be some residual confounding, this suggests that some characteristics related to ethnicity per se may influence care seeking among ethnic minorities. Qualitative studies among Yi and Tibetan women have suggested that local beliefs and fears may lead to women’s suspicion of giving birth in hospital; and cultural inappropriateness of delivery practices may result in women’s discomfort and embarrassment in hospital [39, 46–48]. However, there is dispute over how important cultural barriers to accessing MCH care are, because no uniformity of beliefs and behaviors across population groups have been found [46].

Compared with previous studies [15, 49–52], our study is unique in that we draw ethnic inferences from both geographical and individual comparisons*.* Government reports in China generally make geographical rather than individual comparisons, by, for example comparing autonomous prefectures or counties with prefectures or counties that have a Han majority [21]. Counties designated as autonomous are heterogeneous, however, and such geographic proxy measures may not adequately identify ethnicity. In Sichuan province, for example, the median proportion of ethnic minority people residing in ethnic minority counties is only 66.3%; and in some ethnic minority counties less than half of the population belongs to an ethnic minority. By separating individual ethnicities within ethnic minority counties, we were able to show that Han women living in ethnic minority counties were also disadvantaged compared to those living in majority Han districts and counties. Although less disadvantaged than ethnic minority women, uptake of MCH care was surprisingly low: only a third (32.5%) had received five or more antenatal visits, half (53.8%) gave birth in hospital and two thirds (63.4%) received three doses of DPT.

Our study had some limitations. First, most ethnic minority people in the sample belonged to the Yi and Tibetan ethnic groups, representing only 2 of the 55 ethnic minorities in China. Hence, our results are not representative of all ethnic minorities in China. However, similarly low uptake of MCH care has been reported in western China more generally [5, 49, 53], and our findings are likely to apply to Han and ethnic minority populations with similar socio-economic profiles living in remote mountainous areas. Second, the sampling strategy between 2013 and the other years are inconsistent (four-stage stratified cluster random sampling in 2013 compared to those three-stage strategy in 2003 and 2008). However, we applied multilevel weighted regression models in the analysis to minimize the difference. Third, population-based surveys may be prone to recall bias. This may be particularly important for immunization, where the mother or the caregiver was asked to recall whether or not the child had been immunized. Most investigations into childhood immunization in China rely on immunization cards, health facility records, or both [49, 50, 53]. Poor and uneducated ethnic minority populations may not know whether the child was immunized, although it is not known whether this leads to over- or underreporting of immunization coverage. Fourth, straight line distance to health facilities is a poor representation of actual distance or travel time. Many of the ethnic minority counties included here lie in mountainous terrain, some well above 4000 m. The actual distances that women in ethnic minority counties have to travel may be much greater than the straight line distance that we report here. Fifth, about a third of children had no information on the mother’s education, age and parity, which might have biased the results. Immunization coverage was similarly high among those with or without missing data however, and it is unlikely that this would have biased the results.

## Conclusion

Sichuan province has made considerable efforts to ensure access to MCH care over the last thirty years, underpinned by strong government commitment to and policy enforcement of strategies to reach even the most disadvantaged populations [54]. Despite these efforts, poor ethnic minorities living in remote mountainous areas still fall behind in terms of the national targets of uptake of antenatal care, birth in hospital, caesarean section and DPT immunization. Whilst these populations live far from health facilities and tend to be poorer and less literate, these factors do not fully explain the low uptake of care. Coverage of specific vaccines is much higher than uptake of maternity care, suggesting that bringing services closer to the women with support from health promotion from the township to the village level may encourage more women to seek antenatal care and give birth in hospital. China’s massive efforts in road building and education have also been shown to be a key underlying driver of progress in MCH [2], and this may well be the most important factor to further reduce inequalities in access to MCH care in the future.

## Additional files

Additional file 1: Summary of individual ethnicity in Sichuan Province (2003, 2008, 2013 National Health Service Surveys). (DOCX 22 kb)

Additional file 2: Women’s delivery health facilities in Sichuan Province (2003, 2008, 2013 National Health Service Surveys). (DOCX 16 kb)

## Data Availability

The data that support the findings of this study are available on request from the first author Juying Zhang. The data are not publicly available due to national restrictions on participant privacy.

## Abbreviations

BCG: Bacillus Calmette Guerin
DPT: Diphtheria
MCH: Maternal and child health
