# Supplementary material for: Uptake of maternal care and childhood immunization among ethnic minority and Han populations in Sichuan province: a study based on the 2003, 2008 and 2013 health service surveys
Source: BMC Pregnancy Childbirth. 2019 Jul 16;19:250. doi: 10.1186/s12884-019-2371-y (PMC6636102; doi:10.1186/s12884-019-2371-y)
Supplement: Supplementary file 1 — Summary of individual ethnicity in Sichuan Province (2003, 2008, 2013 National Health Service Surveys). (DOCX 22 kb) [file 12884_2019_2371_MOESM1_ESM.docx]

**Additional file 1**: Summary of individual ethnicity in Sichuan Province (2003, 2008, 2013 National Health Service Surveys)

| **Year** | **Place of residence** | **Ethnicity** | | | | | |
| --- | --- | --- | --- | --- | --- | --- | --- |
|  |  | **Han** | **Tibetan** | **Meng** | **Hui** | **Miao** | **Other** |
| **2003 ^a^** | **Han districts** | 256 | - | - | - | - | 9 |
|  | **Han counties** | 100 | - | - | - | - | 1 |
|  | **Ethnic minority counties** | 25 | - | - | - | - | 137 |
| **2008** | **Han districts** | 204 | - | 1 | 1 | - | 6 |
|  | **Han counties** | 100 | - | - | - | - | - |
|  | **Ethnic minority counties** | 17 | 60 | 4 | 1 | - | 37 |
| **2013** | **Han districts** | 318 | - | 1 | - | 3 | 5 |
|  | **Han counties** | 196 | - | - | - | - | 1 |
|  | **Ethnic minority counties** | 39 | 167 | - | - | - | 65 |

^a^:in 2003 survey year, the individuals were interviewed by Han and other ethnicity, no specific ethnicity minority information were collected.
