# Supplementary material for: Uptake of maternal care and childhood immunization among ethnic minority and Han populations in Sichuan province: a study based on the 2003, 2008 and 2013 health service surveys
Source: BMC Pregnancy Childbirth. 2019 Jul 16;19:250. doi: 10.1186/s12884-019-2371-y (PMC6636102; doi:10.1186/s12884-019-2371-y)
Supplement: Supplementary file 2 — Women’s delivery health facilities in Sichuan Province (2003, 2008, 2013 National Health Service Surveys). (DOCX 16 kb) [file 12884_2019_2371_MOESM2_ESM.docx]

**Additional file 2**: Women’s delivery health facilities in Sichuan Province (2003, 2008, 2013 National Health Service Surveys)

| **Year** | **Place of residence** | **Delivery place** | | | | $\boldsymbol{\chi}^{\boldsymbol{2}}$ | *P* |
| --- | --- | --- | --- | --- | --- | --- | --- |
|  |  | **County hospitals and above** | **Maternal health care (MCH) hospitals** | **Community health centers/Township hospitals** | **Home and other** |  |  |
| **2003** | **Han districts** | 111 | 71 | 34 | 49 | 252.83 | <0.001 |
|  | **Han counties** | 11 | 7 | 38 | 45 |  |  |
|  | **Ethnic minority counties** | 7 | 9 | 7 | 139 |  |  |
| **2008** | **Han districts** | 90 | 78 | 24 | 20 | 222.05 | <0.001 |
|  | **Han counties** | 34 | 13 | 39 | 14 |  |  |
|  | **Ethnic minority counties** | 17 | 3 | 11 | 88 |  |  |
| **2013** | **Han districts** | 149 | 125 | 41 | 12 | 531.46 | <0.001 |
|  | **Han counties** | 101 | 41 | 45 | 10 |  |  |
|  | **Ethnic minority counties** | 38 | 14 | - | 219 |  |  |
